# The Invalidation of *HspB1* Gene in Mouse Alters the Ultrastructural Phenotype of Muscles

**DOI:** 10.1371/journal.pone.0158644

**Published:** 2016-08-11

**Authors:** Malek Kammoun, Brigitte Picard, Thierry Astruc, Mohammed Gagaoua, Denise Aubert, Muriel Bonnet, Véronique Blanquet, Isabelle Cassar-Malek

**Affiliations:** 1 INRA, UMR1213 Herbivores, F-63122, Saint-Genès-Champanelle, France; 2 Clermont Université, VetAgro Sup, UMR1213 Herbivores, BP 10448, F-63000, Clermont-Ferrand, France; 3 INRA, UR0370 Qualité des Produits Animaux, F-63122, Saint-Genès-Champanelle, France; 4 Equipe Maquav, INATAA, Université Frères Mentouri Constantine, Constantine, Algeria; 5 Institut de Génomique Fonctionnelle de Lyon, Université de Lyon, Université Lyon 1, CNRS, INRA, École Normale Supérieure de Lyon, F-69364, Lyon, France; 6 INRA, UMR1061 Génétique Moléculaire animale, F-87060, Limoges, France; University of Maryland Center for Environmental Science, UNITED STATES

## Abstract

Even though abundance of Hsp27 is the highest in skeletal muscle, the relationships between the expression of *HspB1* (encoding Hsp27) and muscle characteristics are not fully understood. In this study, we have analysed the effect of Hsp27 inactivation on mouse development and phenotype. We generated a mouse strain devoid of Hsp27 protein by homologous recombination of the *HspB1* gene. The *HspB1*^-/-^ mouse was viable and fertile, showing neither apparent morphological nor anatomical alterations. We detected a gender dimorphism with marked effects in males, a lower body weight (P < 0.05) with no obvious changes in the growth rate, and a lower plasma lipids profile (cholesterol, HDL and triglycerides, 0.001 < P< 0.05). The muscle structure of the animals was examined by optical microscopy and transmission electron microscopy. Not any differences in the characteristics of muscle fibres (contractile and metabolic type, shape, perimeter, cross-sectional area) were detected except a trend for a higher proportion of small fibres. Different myosin heavy chains electrophoretic profiles were observed in the *HspB1*^-/-^ mouse especially the presence of an additional isoform. Electron microscopy revealed ultrastructural abnormalities in the myofibrillar structure of the *HspB1*^-/-^ mouse mutant mice (e.g. destructured myofibrils and higher gaps between myofibrils) especially in the m. *Soleus*. Combined with our previous data, these findings suggest that Hsp27 could directly impact the organization of muscle cytoskeleton at the molecular and ultrastructural levels.

## Introduction

The heat shock proteins (Hsps) are among the most conserved molecules in phylogeny. They are up-regulated in response to various adverse changes of the environmental conditions inducing cellular stress (e.g. heat shock, nutrient deficiencies, viral infections, various chemicals) [1]. Their up-regulation in stressful conditions is a universal phenomenon, occurring in plant and animal species. In mammals, Hsps are grouped into six main families according to their molecular weight—Hsp110, Hsp90, Hsp70, Hsp60, Hsp47 (Large Hsp)—and the Hsp20 family (Small Hsps, 12–43 kDa). The small Hsp family comprises eleven members (including Hsp27) with various tissue distributions that share a conserved α-crystallin domain [2].

The small Heat shock protein 27 (Hsp27, also denoted Hsp25 in mouse) is constitutively present in a wide variety of tissues and cell lines. Hsp27 belongs to the Hsp chaperone network and plays a key role in the cellular response to stress including heat shock, oxidative and chemical stresses. It is constitutively expressed in small concentrations in the cytosol during unstressed conditions and translocates into or around the nucleus under stress conditions [3]. This protein is encoded by the *HspB1* gene and is transcriptionally regulated by HSF-1. Its post-translational phosphorylation allows for up to 4 isoelectric variants [4]. It is one of the most induced Hsps in response to stress, reaching 1% of the total cellular protein, but accumulates in a slower kinetics than that of Hsp70 the other highly stress responsive Hsp [5]. There is increasing evidences to substantiate Hsp27 as a molecular protector against disease [6] thus used as a biomarker in several disease states [7].

Muscle-specific transcriptional regulatory sequences are found in the *HspB1* gene and the abundance of Hsp27 is the highest in skeletal muscle [8] indicating a crucial role for muscle physiology. *HspB1* expression in muscle is induced by a variety of stimuli [9] *e*.*g*. increased physical activity [10] [11] including lifelong training [12], endurance training [13], and eccentric exercise [14]. Some protective functions of Hsp27 are suggested by its translocation from the cytosolic to myofibrillar fraction and binding to cytoskeletal structures such as Z-disk proteins [15] [14]. The chaperone-like properties of Hsp27 may stabilize myofibrillar proteins during stress conditions and prevent them from loss of function. The relationships between expression of *HspB1* and muscle phenotype have not yet been fully understood apart from a muscle type specific pattern of expression [8]. Hsp27 expression is further detected in zygotes and early embryos [16] and is regulated during mammalian development [17]. To date the role of constitutively expressed Hsp27 is still questioned during development. In this study we have examined the role of constitutive Hsp27 in mouse development and physiology, especially in muscle, by engineering an *HspB1*-null mouse and examining the associated phenotype.

## Material and Methods

### Animals and samples

#### Generation of an HspB1-null mouse

**Fig 1:**
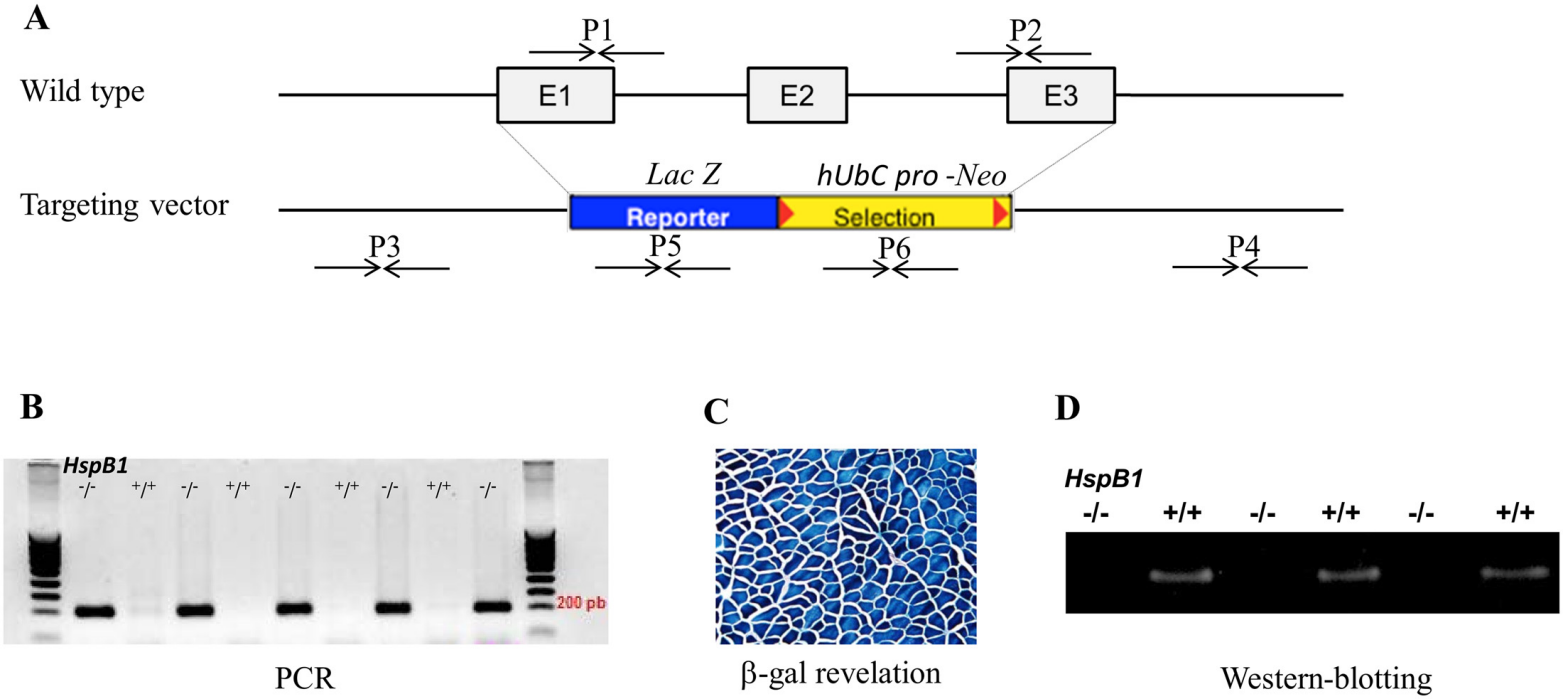
Generation of the *HspB1*^-/-^ -null mouse. (A) The targeting vector was constructed by replacing 1.381 bases of the genomicDNA A, including the three exons of the *HspB1* gene, with a neomycin resistance (*Neo*) gene and *LacZ* reporter gene. The sequences of the primers used are given in Table 1. (B) PCR-based genotyping amplifies targeted construction of *LacZ* reporter gene and *Neo* gene by using primers P5 and P6 respectively. (C) β-gal activity encoded by the *LacZ* reporter gene was used to monitor gene expression in the m. *Tibialis anterior* of the *HspB1*^-/-^ mouse. (D) Absence of Hsp27 protein as confirmed by western-blotting on muscle homogenates prepared from *HspB1*^-/-^ mouse and the wild-type (*HspB1*^+/+^) mouse.

To investigate the role of Hsp27, we generated a constitutive *HspB1*-null mouse using embryonic stem (ES) cells. The cells were provided by the International Knockout Mouse Consortium in which 1.381 bases of the nucleotide sequence of the *HspB1* gene (http://www.velocigene.com/komp/detail/13573) were replaced by a bacterial artificial chromosome (BAC) based vector obtained from BMQ BAC library (Mouse Micer vector set 369N20) (Fig 1A). In the targeting vector, the reporter *LacZ* gene was fused in frame with the start codon of *HspB1* to replace the *HspB1* coding sequence. The mutated ES cells were derived from VGB6 ES cells (C57BL/6NTac background). Three different mutated clones were injected into Balb/c blastocysts to generate 13 chimeras at the SFR Biosciences—Plateau de Biologie Expérimentale de la Souris (PBES, Lyon, France). Male chimeras transmitting the targeted mutation were mated with females from C57BL/6J strain to generate F1 offspring at the experimental facility of nutrition and microbiology of the French National Institute for Agricultural Research (INRA-France). Heterozygous animals (*HspB1*^+/-^) were identified by PCR analysis from tail DNA using different primers as depicted in Table 1. They were inter-crossed to generate homozygous F2 mice (*HspB1*^-/-^) (Fig 1B). The F2 offspring were mated in order to grow the heterozygous and homozygous strains. Data from heterozygous mice with intermediary phenotype between controls and mutants are not given here.

**Table 1:** Primer sequences used for PCR-based genotyping.

Primer pair	Forward sequence	Reverse sequence
P1	CTCGGAGATCCGACAGACG	TCTGGGCAC AAGGGAAACTC
P2	GGGCTGCTTCTGACCTTCTG	CCATGTTCGTCCTGCCTTTC
P3	TCCAGCTACCGGTATTACGC	CTCTGCATT CCTCCCCCTAA
P4	GGTTATCTCTTTTCCCACAG	ATTATGGTGGGCTAGGGATG
P5	TTGACTGTAGCGGGCTGATGTTG	GGTAAACTGGCTCGGATTAGGG
P6	TACTTTCTCGGCAGGAGCAAGGTG	TGGGATCGGCCATTGAACAAGATG

The location of the different primers are presented in Fig 1A

### HspB1-null mice handling

Mouse strains were housed at the technical platform of animal experimentation of UMR 1019 Human Nutrition (Theix, France). They were maintained in a light/dark cycle of 12 hrs, under a controlled room at a temperature of 22°C and humidity of 45–55%. They were fed *ad libitum* with a conventional diet. Experimental procedures and animal handling were done according to the French animal protection legislation including licensing of experimenters. They were reviewed and approved by the French Veterinary Services and the ethics committee, named “Comité Régional d'Ethique en Matière d'Expérimentation Animale Région Auvergne” (agreement number CE 84–12). All efforts were made to minimize suffering. None of the animals utilized for this work became ill or died prior to the experimental endpoint. For plasma and tissue collection, the animals were anesthetized prior to euthanasia by cervical dislocation. Anaesthesia was induced by placing each mouse in an inhalation chamber with 4% isoflurane regulated with calibrated vaporizer.

The mice were weighed once a week during the first 12 postnatal weeks (wk) in order to follow the evolution of their body weight (BW). The average daily gain (ADG) during this period was calculated. Body weight and length were recorded at 8-wk (24 *HspBI*^*-/-*^ and 19 *HspBI*^*+/+*^), 12-wk (25 *HspBI*^*-/-*^ and 20 *HspBI*^*+/+*^) and 52-wk (6 *HspBI*^*-/-*^ and 6 *HspBI*^*+/+*^) of age. The body composition was also determined using the EchoMRI^™^ method that offers measurements of body fat mass and lean mass in live animals.

#### Blood sampling

Blood samples were collected from the retro orbital sinus under general anaesthesia at 4, 8 and 12 wk. Blood were sampled in tubes containing heparin to prevent the formation of clots or extension of existing clots within the blood. Then samples were centrifuged at 4°C (4000 rpm, 10 min) for plasma separation. Plasmas were kept as aliquot fractions at -80°C until analysis.

#### Tissue collection

Mice were sacrificed at 8-wk, 12-wk, and 52-wk of age after blood sampling. Three muscles differing in their fibre types composition were sampled immediately after death, namely the m. *Soleus* (Slow and Fast oxidative), m. *Gastrocnemius* (Fast oxido-glycolytic) and m. *Tibialis anterior* (Fast glycolytic). The liver and three adipose tissues (AT) namely inguinal AT, interscapular AT, and epididymal AT for males were sampled. Tissues were collected at 8-wk, 12-wk, and 52-wk of age. These ages were chosen in order to analyse the effect of Hsp27 inhibition at different stages of development. All the tissues were weighted, frozen in liquid nitrogen for molecular biology analyses or in an isopentane bath cooled by liquid nitrogen for histochemical analyses, and kept at -80°C until use. Ultrastructure analysis, was performed on 2 *HspBI*^*-/-*^ and 2 *HspBI*^*+/+*^mice of 12 weeks old. (*Soleus* and *Tibialis anterior* muscles were removed immediately after death from posterior members of mice. Muscles were pinned to each tendon on a plate cork preserving their lengths before dissection. Muscles were fixed overnight at 4°C by immersion in 2.5% glutaraldehyde in 0.1 M sodium cacodylate buffer pH 7.2.

### Measurements

#### Plasma analyses

The plasma concentrations of 26 parameters representative of muscular, hepatic, lipidic, and energetic metabolism (urea, glucose, amylase, uric acid, phosphorus, calcium, cholesterol, triglycerides, low-density lipoprotein (LDL), high-density lipoprotein (HDL), cholinesterase, total bilirubin, total proteins, alkaline phosphatase, alanine aminotransferase, aspartate aminotransferase, iron, albumin, creatinine, creatine kinase, creatine kinase MB, lactate, lactate dehydrogenase, magnesium, transferrin and hydroxybutyrate dehydrogenase) were measured using a KONELAB 30 automat (Thermo scientific, Cergy Pontoise, France) at the Faculty of Science and Technology of Limoges (France) as described by Magnol *et al*. [18].

#### Histology, immunohistochemical and β-galactosidase staining

Serial sections of 10 μm thick, were cut on a cryostat (Cryo-star HM 560 Microm International GmbH, Germany) at -26°C, perpendicular to the muscle fibres. Adjacent sections were used for immunohistochemical and histochemical analyses and for β-galactosidase staining.

A semi-automatic classification of fibres was used for classification of muscle fibre by using a combination of only two anti- Myosin heavy chain (MyHC) antibodies [19]. For m. *Soleus* BA-D5 (specific of slow MyHC I) and SC71 (specific of IIa MyHC) were used on serial sections. For m. *Tibialis anterior* BF-F3 specific of IIb MyHC and SC71 were used on serial sections according to Kammoun *et al*. [19]. The anti-MyHC antibodies were purchased from AGRO-BIO (La Ferté Saint-Aubin, France). After immuno-staining of MyHC isoforms, histological sections were visualized under an Olympus fluorescence microscope BX 51, using a 10X objective (NA = 0.3) and adequate band pass filter (Alexa 488: excitation filter 460–495, emission filter 510–550, dichromatic mirror 505LP; Cy3: excitation filter 530–550, emission filter 575–625, dichromatic mirror 565LP) as described by Meunier *et al*. [20]. This technique allowed us to determine the muscle fibre type proportions, meaning cross-sectional for an average 300 fibres per serial image. The metabolic fibre type was determined by revealing the succinate dehydrogenase (SDH, a mitochondrial enzyme representative of oxidative metabolism) activity according to Ashmore [21].

For *HspB1*^-/-^ mice muscles, serial sections were also stained for β-galactosidase (β-gal) activity in order to detect reporter gene expression. Individual images were assembled into composite panoramic images of MyHC analysis, SDH activity and β-gal staining.

#### Electrophoretic separation of MyHC isoforms

Myofibrillar proteins were extracted using a buffer containing 0.5 M NaCl, 20 mM NaPPi, 50 mM Tris, 1 mM EDTA and 1 mM DTT according to the protocol described by Picard *et al*. [22]. After homogenization using a Polytron (x22 000), the muscle extract was subjected to a centrifugation at 2500 x g for 10 min at 4°C. Then the supernatants were diluted 1:1 (v/w) with glycerol and stored at -20°C until use. The protein concentrations of the extracts were determined according to the Bradford method [23] using the Bio-Rad Protein assay. Bovine serum albumin (BSA) at a concentration of 1 mg/mL was used as standard.

The samples were suspended in 1:1 v/v in a basic 2 x Laemmli solution containing 4% w/v SDS, 10% v/v β-mercaptoethanol, 20% v/v glycerol, 125 mM Tris (pH 6.8) and 0.01% w/v pyronin Y, incubated at room temperature for 10 min and then heated (70°C) for 10 min. The MyHC isoforms were separated with SDS glycerol gel electrophoresis according to Picard *et al*. [22]. The stacking gel contained 4% polyacrylamide with a cross-link of 1.96%. The separating gel was an 8% polyacrylamide gradient with a cross-link of 1%. Three micrograms of protein extracts were loaded per well onto 0.75-mm-thick gels. The electrophoresis was run a Mini-PROTEAN^®^ II vertical chamber (Bio-Rad, USA) at a constant voltage of 140V for 15 hours at 4°C. After staining in a Coomassie Blue dye solution, the gels were scanned using the Expression 10000XL Pro scanner (Epson) and the proportions of the different MyHCs bands were quantified by densitometry with ImageQuant Software 5500 (Amersham Biosciences/GE Healthcare).

#### Western-blot analyses

Invalidation of *HspB1* gene was validated in the muscle of *HspB1*^-/-^ *vs* controls using a quantitative western-blotting technique as described by Chaze *et al*. [24]. Fifteen or thirteen μg of total protein extracts were separated by gel electrophoresis using SDS-PAGE [25]. After migration, the proteins were transferred onto PVDF membrane (Millipore, Bedford, MA01730 U S A). Membranes were blocked using a TBS 1x buffer containing 5% skimmed milk and incubated under gentle agitation overnight at room temperature in the presence of the Hsp27 primary antibody (Santa Cruz: SC13132; 1/1000). The morning, the membranes were incubated at 37°C for 30 min. with the secondary fluorochrome-conjugated LICOR-antibody. Infrared fluorescence detection was then used for protein quantification Membranes were scanned by the scanner Odyssey (LI-COR Biosciences) at 800 nm. Band volumes were quantified in the images using ImageQuant TL v 2003 software (Amersham).

#### Ultrastructure and image analysis

Small blocks (1 to 3 mm^3^ with fibre direction identified) were prepared from the glutaraldehyde fixed muscles and were post-fixed in 1% osmium tetroxide in 0.1 M SCB with adapted pH (7.2 or 5.8) for 1 hr at room temperature. The blocks were dehydrated through a graded series of ethanol and embedded in epoxy resin (TAAB, Eurobio France) by using an automatic microwave tissue processor for electron microscopy (EM AMW, Leica company,Vienna, Austria). Ultra-thin sections (90 nm) were stained with uranyl acetate and lead citrate and observed using a transmission electron microscope (Hitachi HM 7650) at 80 kV acceleration voltage. Micrographs were obtained using a Hamamatsu AMT digital camera system coupled to the microscope. All samples were prepared and observed at the Cellular Imaging Center for Health (CICS) lab at Clermont-Ferrand University (France).

The width of myofibrils and the intermyofibrillar spaces were determined on the images of soleus and tibialis longitudinal sections of 12 weeks old mice using the open source ImageJ software (http://rsb.info.nih.gov/ij/). The grey scale ranges extend from 0 (black) to 255 (white). The image analysis included automatic thresholding, and morphological measuring. For each numerical object labelled the area estimation was based on pixels number count, the coloration intensity corresponded on an 8 bit grey level mean value. The analysis was performed on a total of 210 images distributed in 96 images for wild-type (n = 2 mice) and 114 images for *HspB1*^-/-^ mice (n = 2). Four fields were analysed per image and therefore the measurements were performed in 480 electron microscopy fields.

#### Statistical analysis

Statistical analyses were performed using the PROC GLM procedure (SAS Inst. Inc., Cary, NC) and mean comparisons by using post hoc procedures (Newman-Keuls) were performed to analyse differences (*P* < 0.05). The first model tested fixed effects of genotype, age, and gender, and all interactions for growth, body composition, tissue weights and plasma chemistry parameters. The second model tested fixed effects of genotype, muscle, and gender, and all interactions for muscle ultrastructure. Least squares means were generated for all interactions and main effects. For electronic microscopy micrographs the statistical analysis was carried out according to [26] using a two-way ANOVA with repeated measures followed by multiple comparisons *post-hoc* Tukey correction for analyzing the interactions. The analyses were performed using the repeated measure option of the PROC GLM of SAS software, and results were considered significant at p < 0.05. Values are presented as means ± sem.

## Results

### Broad general phenotyping of the *HspB1*-null mouse

The generated *HspB1*^-^/^-^ mice were viable, fertile and born after 21 days of gestation at the expected Mendelian distribution. Revelation of β-gal activity in muscle confirmed that the endogenous *HspB1* promoter driven construction was expressed in the *HspB1*^-/-^ mice (Fig 1C). Western-blotting analysis of muscles homogenates confirmed the absence of Hsp27 (Fig 1D).

The *HspB1*^-^/^-^ mutant mice showed no apparent morphological or anatomical alterations compared to homozygous controls until up to 18 months. The weights of new born mice were lower compared to controls (1.3 g for *HspB1*^-/-^ *vs* 2 g for *HspB1*^+/+^, *P* < 0.001) at one day after birth. As expected, during postnatal development (S1 Dataset), there was a significant effect of age (0.001 < *P* < 0.01, Table 2) and of gender (0.001 < *P* < 0.05, Table 2) on growth and body composition parameters however a genotype effect was observed only for body weight (BW) (*P* < 0.05, Table 2). There was no genotype effect on tissue weights expressed as a proportion of animal BW (Table 2). Significant interactions were detected and reported in Table 2 for BW and relative weight of fat (gender × genotype, *P* < 0.05) liver and m. *Soleus* tissues (age × gender × genotype, *P* < 0.05).

**Table 2:** Influence of *HspB1* gene disruption on growth parameters, body composition and relative tissue weights ^1^.

Main effect	BW, g	Length, cm	Fat, mg/g	Lean mg/g	Liver mg/g	m. *Soleus* mg/g	m. *Gastrocnemius* mg/g	m. *Tibialis anterior* mg/g	AT. *Epididymal* mg/g	AT. *Inguinal* mg/g	AT. *Interscapular* mg/g
**Genotype (Ge)** ^2^											
*HspB1*^+/+^ (n = 45)	23.20	6.71	9.74	85.93	5.64	0.07	0.87	0.31	1.29	0.79	0.65
*Male (n = 25)*	*25*.*24*	*6*.*85*	*9*.*14*	*86*.*42*	*5*.*83*	*0*.*07*	*0*.*89*	*0*.*31*	*1*.*29*	*0*.*74*	*0*.*62*
*Female (n = 20)*	*20*.*64*	*6*.*53*	*10*.*50*	*85*.*32*	*5*.*39*	*0*.*07*	*0*.*86*	*0*.*32*	*/*	*0*.*84*	*0*.*69*
*HspB1*^-/-^ (n = 55)	22.0	6.59	9.22	86.66	5.40	0.07	0.89	0.35	1.21	0.75	0.60
*Male (n = 33)*	*23*.*43*	*6*.*71*	*8*.*15*	*87*.*71*	*5*.*40*	*0*.*07*	*0*.*92*	*0*.*37*	*1*.*21*	*0*.*68*	*0*.*55*
*Female (n = 22)*	*19*.*84*	*6*.*41*	*10*.*84*	*85*.*08*	*5*.*39*	*0*.*07*	*0*.*84*	*0*.*32*	*/*	*0*.*86*	*0*.*67*
*P*-value ^3^	*	ns	ns	ns	ns	ns	ns	ns	ns	ns	ns
**Gender (G)**											
Male (n = 58)	24.21	6.77	8.57	87.15	5.59	0.07	0.90	0.35	1.24	0.71	0.58
Female (n = 42)	20.22	6.46	10.68	85.20	5.39	0.07	0.85	0.32	/	0.85	0.68
*P*-value	***	**	**	*	ns	ns	ns	ns	/	**	*
**Age (A)** ^4^											
8 wk (n = 43)	19.59^c^	6.36^c^	7.75^b^	88.06^a^	5.80^a^	0.07^a,b^	0.88	0.37	0.87^c^	0.67^b^	0.58^b^
12 wk (n = 45)	23.03^b^	6.61^b^	7.95^b^	87.65^a^	5.44^a^	0.074^a^	0.90	0.32	1.18^b^	0.67^b^	0.53^b^
52 wk (n = 12)	31.25^a^	7.71^a^	21.23^a^	75.17^b^	4.80^b^	0.065^b^	0.83	0.29	3.13^a^	1.46^a^	1.11^a^
*P*-value	***	***	***	***	**	**	ns	ns	***	***	***
**Interactions**											
Ge x G	*	ns	*	ns	ns	ns	ns	ns	/	ns	ns
Ge x A	**	ns	ns	ns	ns	ns	ns	ns	ns	ns	ns
G x A	***	ns	ns	ns	ns	ns	ns	ns	/	**	ns
Ge x G x A	*	ns	ns	ns	*	*	ns	ns	/	ns	ns

^1^ Muscle weight, adipose tissues (AT) weight, Liver weight, Fat and Lean are relative to animal body weight.

^2^ *HspB1*^-/-^: *HspB1*-null mouse; *HspB1*^+/+^: Wild type littermates.

^3^ Significance: *: *P* < 0.05; **: *P* < 0.01; ***: *P* < 0.001; ns: not significant.

^4^ LS-means with different superscripts (a, b, c) within a column for age effect are significantly different (*P* < 0.05).

**Fig 2:**
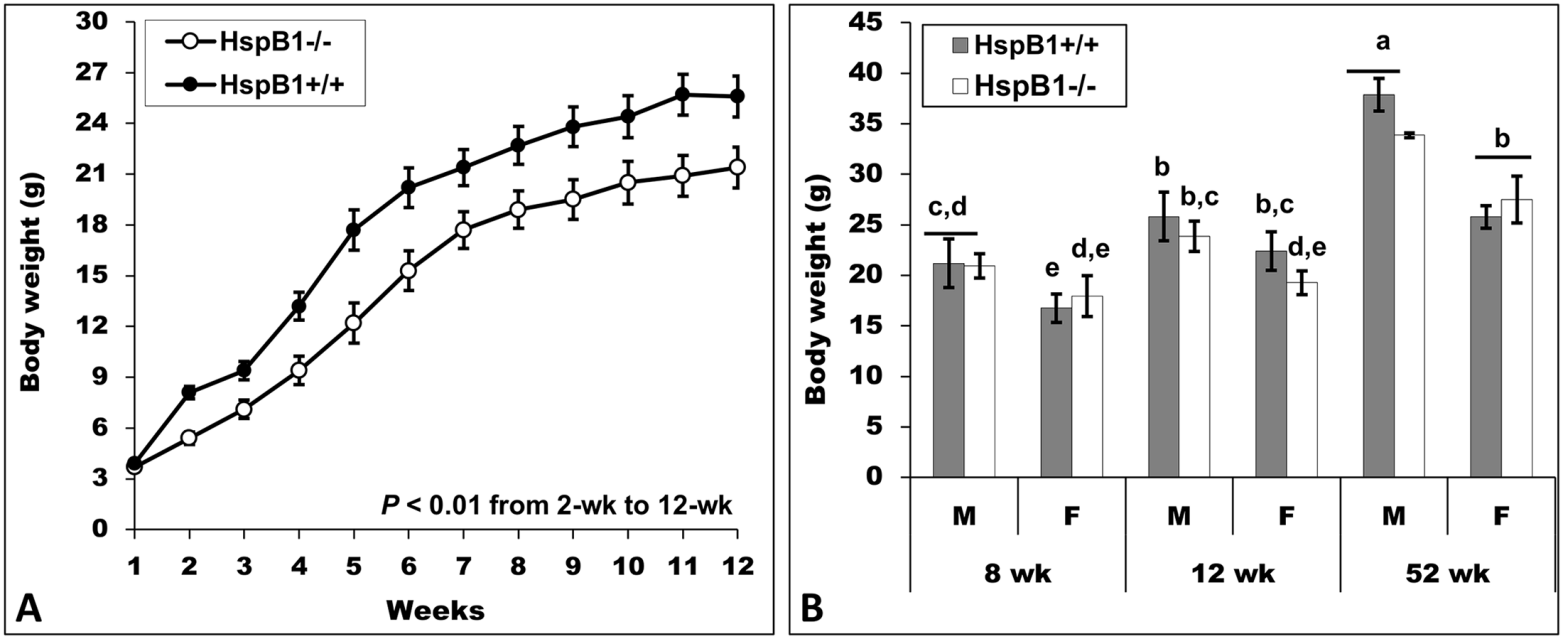
Influence of *HspB1* gene disruption on the growth and size of animals. (A) Evolution of body weight from 1 to 12 weeks of age. (B) Body weight at 8-wk, 12-wk, and 52-wk of age; *HspB1*^-/-^: *HspB1*-null mice; *HspB1*^+/+^: Wild type littermates; F: Females; M: Males. LS-means (± SD) with different superscripts (^a, b, c, d, e^) are significantly different (*P* < 0.05).

Fig 2A illustrates BW evolution from 1 to 12 weeks of age in both genotypes. The BW of *HspB1*^-^/^-^ mice were significantly lower than that of controls from week 2 to 12 of age (*P* < 0.01) but the average daily gain (data not shown) did not differ between the two groups (0.21 to 0.23, *P* > 0.05). The *HspB1*^-^/^-^ males were lighter from 12-wk-old onwards (Fig 2B). Similar differences were recorded for females only at 12- wk of age. No differences were observed in length at the three ages analysed.

Of the 26 plasma parameters measured (Table 3), significant differences were detected between genotypes for the lipids profile (cholesterol, HDL, and triglycerides, 0.001 < *P* < 0.05), total bilirubin (*P* < 0.05), calcium (*P* < 0.01) and iron (*P* < 0.05). The data showed a decrease in lipidic metabolites for the *HspB1*-null mouse. Significant age × gender × genotype interactions (0.01 < *P* < 0.05) were also detected for glucose, total bilirubin and aspartate aminotransferase (Table 3) indicating that the liver functions (probably lipogenesis and detoxification) has been likely affected by *HspB1* gene invalidation.

**Table 3:** Influence of *HspB1* gene disruption on the plasma chemistry parameters during postnatal growth.

**A**													
**Main effect**	**Albumin g/L**	**Transferrin g/L**	**Alkaline phosphatase UI/L**	**Alanine aminotransf. UI/L**	**Aspartate aminotransf. UI/L**	**Total bilirubin μmol/L**	**Amylase UI/L**	**Cholinesterase UI/L**	**Creatine Kinase C UI/L**	**Creatine Kinase MB, UI/L**	**Creatinine UI/L**	**Uric acid μmol/L**	**Urea mmol/L**
**Genotype (Ge)** ^1^													
*HspB1*^*+/+*^ (n = 30)	27.0	1.01	124.52	41.51	105.7	1.24	2742.6	1452.8	439.5	396.6	21.86	78.86	9.84
*Male (n = 18)*	*26*.*64*	*1*.*00*	*121*.*14*	*48*.*96*	*101*.*73*	*1*.*11*	*2914*.*14*	*1274*.*1*	*439*.*4*	*387*.*9*	*20*.*97*	*79*.*14*	*9*.*76*
*Female (n = 12)*	*27*.*54*	*1*.*02*	*153*.*29*	*30*.*33*	*111*.*62*	*1*.*42*	*2485*.*30*	*1721*.*0*	*439*.*7*	*409*.*6*	*23*.*20*	*78*.*45*	*9*.*97*
*HspB1*^*-/-*^ (n = 27)	26.63	1.00	119.25	43.10	107.4	1.48	2812.2	15.28.2	460.9	348.1	21.52	77.25	9.40
*Male (n = 15)*	*26*.*29*	*1*.*01*	*116*.*49*	*55*.*70*	*101*.*53*	*1*.*63B*	*2986*.*60*	*1320*.*5*	*368*.*9*	*351*.*9*	*21*.*35*	*71*.*69*	*8*.*80*
*Female (n = 12)*	*27*.*05*	*0*.*99*	*134*.*77*	*27*.*35*	*114*.*68*	*1*.*29*	*2594*.*33*	*1787*.*7*	*575*.*8*	*343*.*5*	*21*.*74*	*84*.*19*	*10*.*14*
*P*-value ^2^	ns	ns	ns	ns	ns	*	ns	ns	ns	ns	ns	ns	ns
**Gender (G)**													
Male (n = 33)	26.5	1.01	106.63	52.02	101.64	1.35	2947.1	1295.2	507.8	371.5	21.14	75.75	9.33
Female (n = 24)	27.3	1.00	137.15	28.84	113.15	1.35	2539.8	17.54.3	407.4	376.5	22.50	81.32	10.06
*P*-value	ns	ns	***	***	ns	ns	***	***	ns	ns	ns	ns	*
**Age (A)** ^3^													
8 wk (n = 24)	26.40	0.98	169.07^a^	22.1^b^	104.5^b^	1.27	2608.5^b^	1295.7^c^	542	408.5	21.41	75.78	9.41^b^
12 wk (n = 21)	27.16	1.04	112.90^b^	35.14^b^	94.90^b^	1.43	2677.7^b^	1527.1^b^	426	318.9	23.54	79.89	9.09^b^
52 wk (n = 12)	27.13	1.00	79.40^c^	95.02^a^	130.8^a^	1.36	3281.1^a^	1806.5^a^	486	397.7	19.07	79.58	11.04^a^
*P*-value	ns	ns	***	***	*	ns	***	***	ns	ns	ns	ns	**
**Interactions**													
Ge x G	ns	ns	ns	ns	ns	***	ns	ns	ns	ns	ns	ns	ns
Ge x A	ns	ns	*	ns	ns	ns	*	**	ns	ns	ns	ns	*
G x A	ns	ns	ns	**	ns	ns	**	**	ns	ns	ns	ns	*
Ge x G x A	ns	ns	ns	ns	*	**	ns	ns	ns	ns	ns	ns	ns
**B**													
**Main effect**	**Iron μmol/L**	**Magnesium mmol/L**	**Phosphorus mmol/L**	**Calcium mmol/L**	**Glucose mmol/L**	**Lactate μmol/L**	**Lactate dehyd. UI/L**	**hydroxybutyrate dehyd. UI/L**	**Cholesterol mmol/L**	**HDL mmol/L**	**LDL mmol/L**	**Triglyceride. mmol/L**	
**Genotype (Ge)**													
*HspB1*^*+/+*^ (n = 30)	26.71	0.81	2.59	2.28	12.32	6308.1	1042.4	169.4	2.36	1.69	0.22	1.10	
*Male (n = 18)*	*26*.*94*	*0*.*79*	*2*.*66*	*2*.*30*	*12*.*42*	*6459*.*8*	*1047*.*3*	*167*.*6*	*2*.*57*	*1*.*86*	*0*.*19*	*1*.*23*	
*Female (n = 12)*	*26*.*38*	*0*.*85*	*2*.*49*	*2*.*24*	*12*.*18*	*6082*.*2*	*1035*.*1*	*172*.*2*	*2*.*05*	*1*.*44*	*0*.*27*	*0*.*90*	
*HspB1*^*-/-*^ (n = 27)	23.71	0.80	2.75	2.04	11.69	6020.1	884.5	153.9	2.08	1.49	0.21	0.91	
*Male (n = 15)*	*22*.*69*	*0*.*77*	*2*.*85*	*2*.*06*	*11*.*80*	*6220*.*1*	*936*.*8*	*159*.*7*	*2*.*25*	*1*.*68*	*0*.*18*	*0*.*87*	
*Female (n = 12)*	*24*.*99*	*0*.*84*	*2*.*63*	*2*.*00*	*11*.*54*	*5771*.*9*	*819*.*0*	*146*.*8*	*1*.*86*	*1*.*27*	*0*.*26*	*0*.*95*	
*P*-value	*	ns	ns	**	ns	ns	ns	ns	**	***	ns	**	
**Gender (G)**													
Male (n = 33)	25.00	0.78	2.74	2.19	12.14	6351	997.1	164	2.42	1.76	0.18	1.07	
Female (n = 24)	25.70	0.85	2.56	2.12	11.86	5927	927.1	160	1.96	1.35	0.26	0.92	
*P*-value	ns	**	ns	ns	ns	ns	ns	ns	***	***	**	**	
**Age (A)**													
8 wk (n = 24)	26.40	0.81	2.94^a^	2.20	12.86^a^	6073.3^b^	891.2^b^	153.12	2.20	1.57	0.25^a^	0.99^b^	
12 wk (n = 21)	24.50	0.80	2.64^b^	2.07	12.10^a^	5529.2^b^	926.4^b^	150.63	2.22	1.62	0.21^a,b^	0.88^b^	
52 wk (n = 12)	24.64	0.83	2.17^c^	2.24	10.28^b^	7496.2^a^	1192.4^a^	200.12	2.28	1.60	0.16^b^	1.24^a^	
*P*-value	ns	ns	***	ns	***	**	*	ns	ns	ns	**	***	
**Interactions**													
Ge x G	ns	ns	ns	ns	ns	ns	ns	ns	ns	ns	ns	***	
Ge x A	ns	ns	**	ns	**	ns	ns	ns	ns	ns	ns	ns	
G x A	ns	*	ns	ns	ns	ns	ns	ns	**	*	ns	ns	
Ge x G x A	ns	ns	ns	ns	*	ns	ns	ns	ns	ns	ns	ns	

Abbreviation: aminotransf: aminotransferase; dehyd: dehydrogenase

^1^ *HspB1*^-/-^: *HspB1*-null mouse; *HspB1*^+/+^: Wild type littermates.

^2^ Significance: *: *P* < 0.05; **: *P* < 0.01; ***: *P* < 0.001; ns: not significant.

^3^ LS-means with different superscripts (a, b, c) within a column for age effect are significantly different (*P* < 0.05).

### Muscle changes associated with Hsp27 deficiency

#### Muscle properties

**Fig 3:**
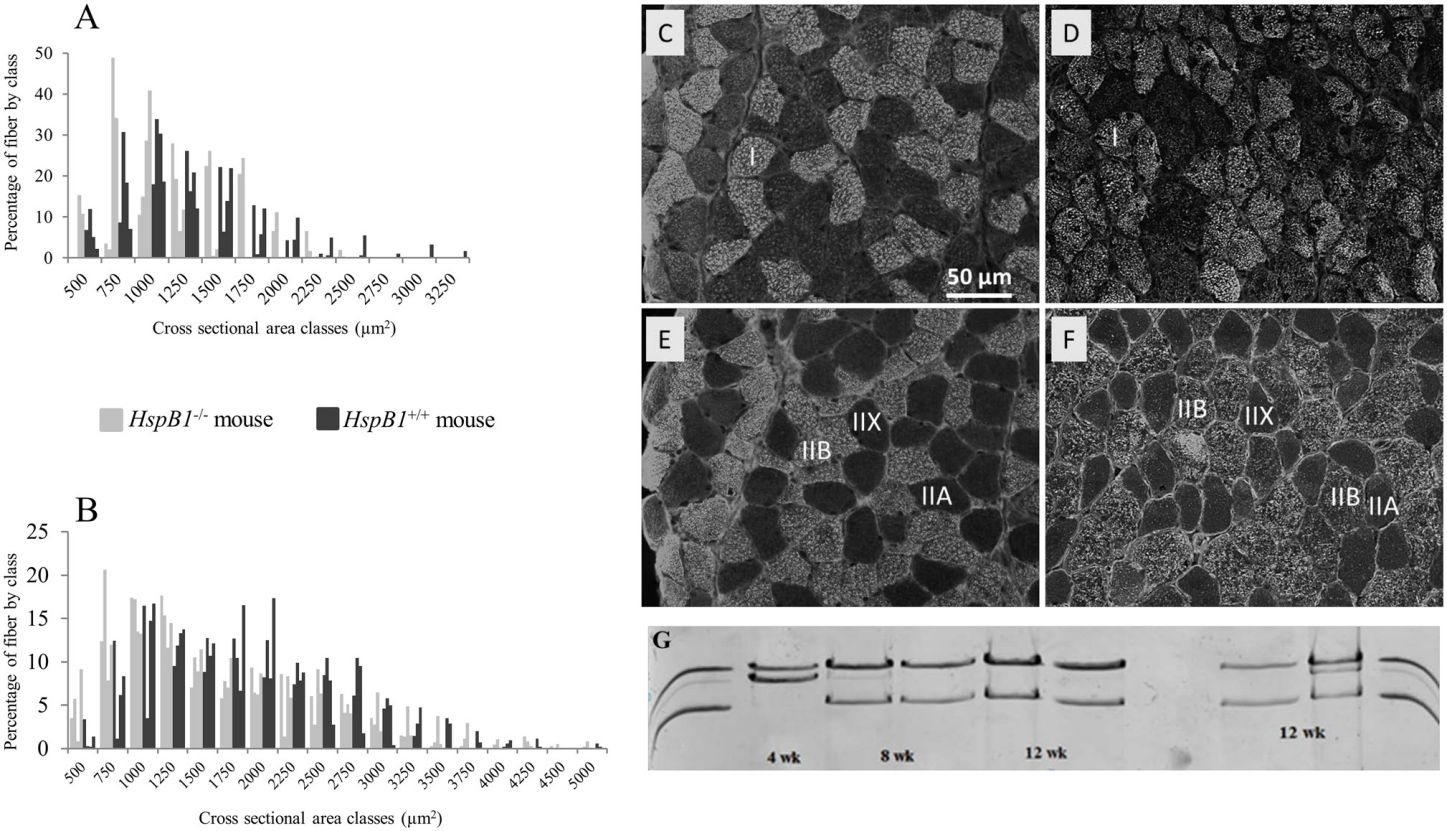
Influence of *HspB1* gene invalidation on muscle fibre characteristics. (A, B) Frequency profiles of muscle fibre cross sectional area at 12 weeks of age in the m. *Soleus* (A) and the m. *Tibialis anterior* (B). (C-F) Myosin heavy chain (MyHC) immunostaining in the m. *Soleus* (C, D, BAD5 staining) and m. *Tibialis anterior* (E, F, BF-F3 immuno-staining) at 12 weeks of age. (C, E) wild type controls; (D, F) *HspB1*^-/-^ mice. Fibre types: I, slow oxidative fibre; IIA and IIX, fast oxido-glycolytic fibre; IIB, fast glycolytic fibre. *Scale bar*: *50 μm*; (G) electrophoretic separation of MyHC at 3 ages in wild type controls (*HspB1*^+/+^) and *HspB1*^-/-^ mutant mice.

As illustrated in Table 2, the skeletal muscles analysed, whether oxidative or glycolytic types, showed no differences with respect to weights between *HspB1*^-/-^ mice and wild-types. To investigate muscle phenotyping, we focused on 12-wk old adult males. Examination of fibre types (Fig 3) showed that the expression of MyHC isoforms, cross-sectional areas, shapes and perimeters of fibres did not differ between genotypes in the *Soleus* and *Tibialis anterior* muscles (Fig 3C–3F). However the frequency profiles of fibre sizes were different between genotypes, with a tendency to higher numbers of small fibres in the *HspB1*^-/-^ mouse than wild-type for both *Soleus* and *Tibialis anterior* muscles (Fig 3A and 3B). SDH activity, an indicator of mitochondria content, revealed no obvious differences between the *HspB1*^-/-^ mouse and wild-type mouse (data not shown). In the muscles of *HspB1*^-/-^ mouse, expression of the *HspB1* promoter driven construction was found to be fibre-type specific (type IIB < type IIA < type I and IIX) (Fig 1 and data not shown). An adequate electrophoresis technique to quantify proportions of MyHC revealed distinct profiles between the *HspB1*^-/-^ mouse and the wild-type mouse in the m. *Soleus* (Fig 3G right panel). A third MyHC was detected in *HspB1*^-/-^ mice comparatively to their controls. As illustrated in Fig 3G (left panel) this MyHC could be similar to the developmental isoform detected at 4 weeks of age in the wild-type mouse. However, this could not be validated by immunohistochemistry likely because the fast antibody cross-reacted with developmental MyHC [27].

#### Muscle ultrastructure

**Fig 4:**
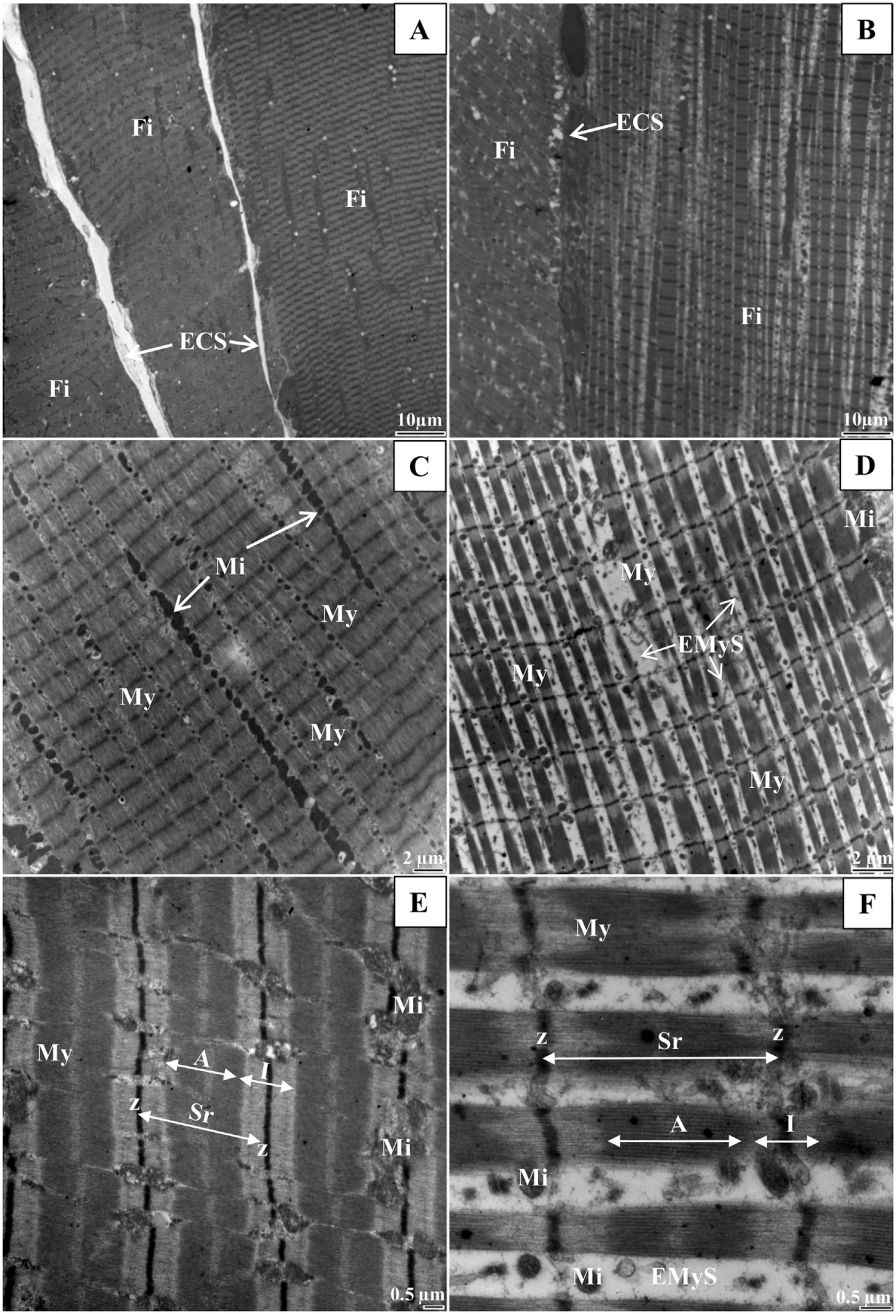
The loosely ultrastructure of the *Soleus* muscle associated with invalidation of Hsp27. (A, C, and E): *HspB1*^+/+^ (Wild type control); (B, D, and F): *HspB1*^-/-^ (*HspB1*-null mouse). Fi: muscle fibre, ECS: extra cellular space, My: Myofibril, EMyS: Extramyofibrillar space, Mi: Mitochondria, Sr: Sarcomere, A: A band, I: I band.

**Fig 5:**
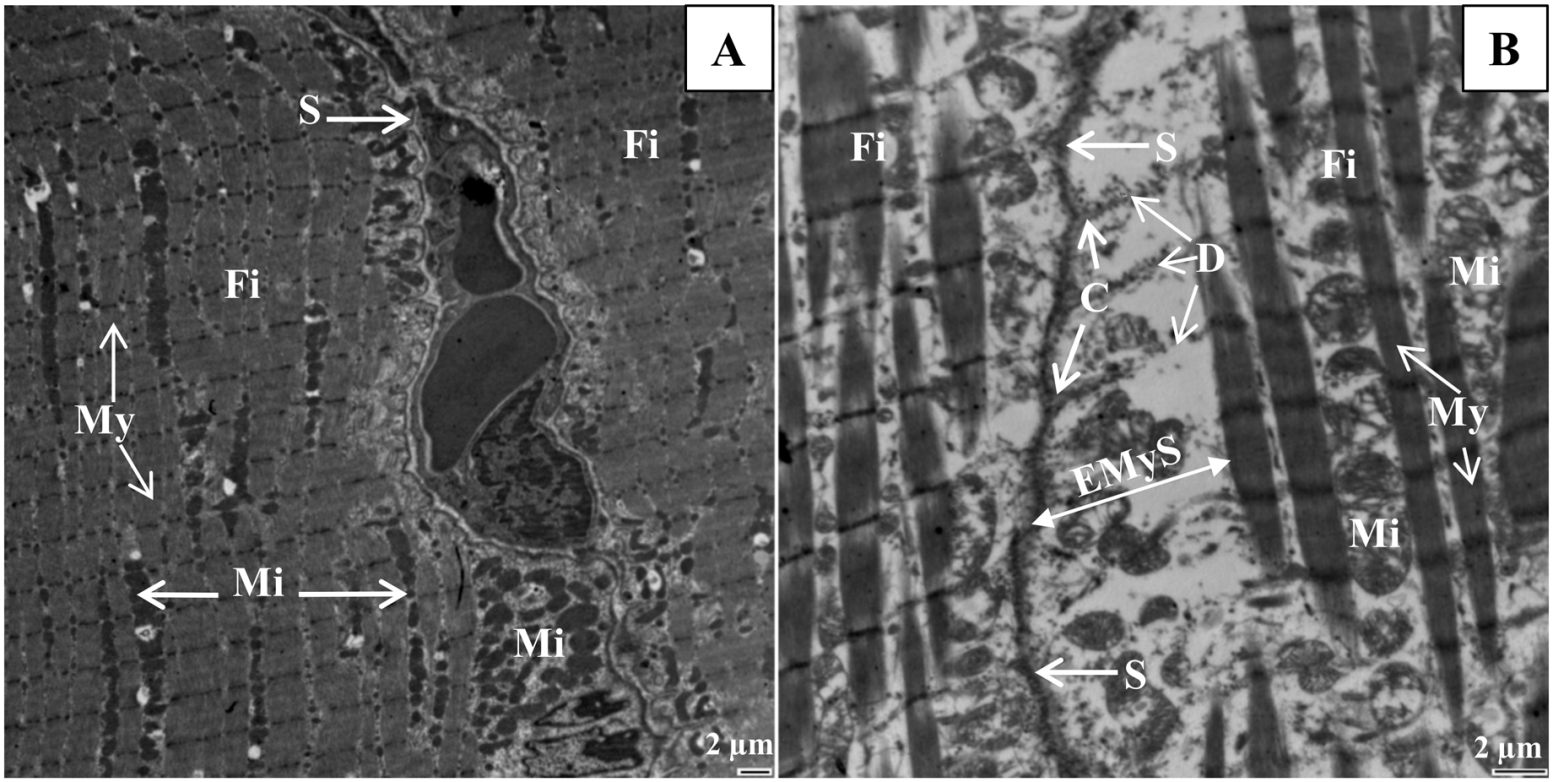
Details of the ultrastructure of *Soleus* muscle of the *HspB1*^-/-^ mouse. Desmin lateral attachments between myofibrils to the sarcolemma are shown in the *HspB1*-null mouse. (A) Well defined sarcolemma in the wild type control; (B) disintegration of the lateral attachments between myofibrils to the sarcolemma. Fi: muscle fibre, My: Myofibril, Mi: Mitochondria, C: Costamers, D: Desmin Filaments, S: Sarcolemma, EMyS: Extramyofibrillar space.

**Fig 6:**
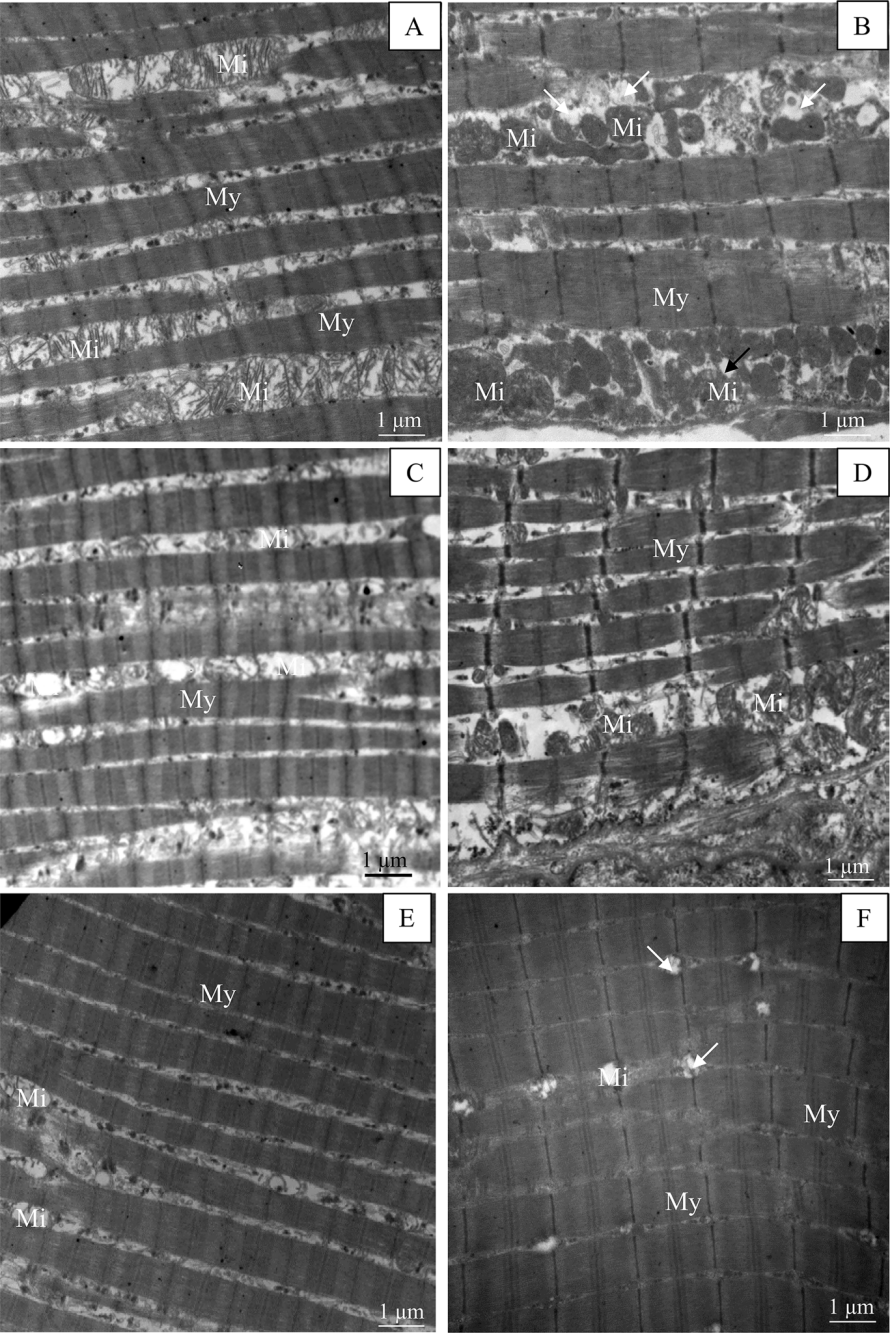
Ultrastructure of the *Tibialis anterior* muscle of the *HspB1*^-/-^ mouse. (A, C, and E): *HspB1*^+/+^ (Wild type mouse); (B, D, and F): *HspB1*^-/-^ (*HspB1*-null mouse). My: Myofibril. Arrows: Altered mitochondria. (A, B, C, D): oxidative fibres characterized by high mitochondria content, (E, F) glycolytic fibres with small amount of mitochondria.

To examine the in-depth phenotype of muscle, we performed electron microscopy of longitudinal muscle sections and measurements. Figs 4–6 illustrate changes in muscle ultrastructure following Hsp27 invalidation. Compared to the wild-type muscle (Fig 4A, 4C and 4E), the soleus muscle of the *HspB1*^-/-^ mouse (Fig 4B, 4D and 4F) showed increased intermyofibrillar spaces (Table 4) and altered sarcomere (longitudinally repeating subunit of myofibrils) structure with Z lines deformation, A band and I band junctions not well defined. The sarcomere length (1.70 μm ± 0.3) was not changed. The costamers were stretched (Fig 5B *vs*. 5A). Some disintegration of the lateral attachments between myofibrils and the sarcolemma were also observed (Fig 5B). The morphology of nuclei appeared to be normal. They were localized at the periphery of myofibrils (data not shown). There appeared to be no differences in mitochondria numbers between *HspB1*^-/-^ mice and controls (Fig 4C and 4D). However, an alteration was observed in the morphology of some mitochondria in *HspB1*^-/-^ muscles. Several fibres showed unaltered ultrastructure suggesting a high heterogeneity in the ultrastructure with altered and non-altered regions (Fig 4).

**Table 4:** Influence of *HspB1* gene disruption on muscle ultrastructure.

	Genotype (Ge) ^1^		Gender (G)		Muscle (M)		SEM ^3^	Significance ^2^						
	*HspB1*^*+/+*^	*HspB1*^*-/-*^	Female	Male	*m*. *Soleus*	*m*. *Tibialis anterior*		Ge	G	M	Ge x G	Ge x M	G x M	Ge x G x M
Average width of the intermyofibrillar spaces ^4^ (nm)	227.5 (14.11)	275.2 (19.74)	200.7 (11.43)	302.0 (18.48)	284.0 (20.50)	218.7 (14.81)	12.22	*	***	**	**	***	**	**
Percentage occupied by the intermyofibrillar spaces (nm)	21.3 (0.91)	24.7 (1.42)	19.2 (0.89)	26.8 (1.20)	25.0 (1.68)	21.0 (0.91)	0.85	**	***	**	***	***	**	***
Average width of the myofibrils (nm)	770.0 (24.53)	754.7 (19.64)	754.3 (20.46)	770.4 (23.25)	814.5 (30.16)	710.2 (16.01)	15.60	ns	ns	***	***	*	ns	ns

The measurements were performed in 480 electron microscopy fields.

^1^ *HspB1*^-/-^: *HspB1*-null mouse; *HspB1*^+/+^: Wild type littermates.

^2^ Significance: *: *P* < 0.05; **: *P* < 0.01; ***: *P* < 0.001; ns: not significant.

^3^ SEM: standard error of the mean.

^4^ LS-means (± SD).

The ultrastructure of the m. *Tibialis anterior* in the mutant mouse is presented in Fig 6. Four fibre types (I, IIA, IIB and IIX) are observed. The oxidative fibres (Fig 6A, 6B, 6C and 6D) are characterized by a high content of mitochondria while glycolytic fibres (Fig 6E and 6F) are poor in mitochondria. Changes in m. *Tibialis anterior* ultrastructure were less marked than in the *Soleus* muscle. Glycolytic fibres showed very small intermyofibrillar spaces and their ultrastructure was apparently not affected in the mutant mouse. *HspB1*^-/-^ oxidative fibres exhibited larger space between myofibrils than controls (Table 4). Lastly *HspB1*^-/-^ fibres showed more altered mitochondria.

## Discussion

Small heat shock proteins (sHsps) play a fundamental role in multiple organisms through pleiotropic and more specific effects [28]. They are tightly regulated during development [17]. To investigate the role of constitutive Hsp27 *in vivo*, we generated a mouse strain carrying targeted invalidation of the *HspB1* gene. We examined the consequences of Hsp27 deficiency on the broad phenotype of mutant mouse, and specifically on muscle development and fine characteristics. The final objective was to look for molecular mechanisms regulated by Hsp27 that could account for muscle development, structure, and physiology.

The invalidation of *HspB1* appeared to have only a moderate effect on the development and physiology since the mutant mice were viable, fertile and apparently normal offspring were generated. Since Hsp27 is constitutively present in a wide variety of tissues as part of the chaperone network, the occurrence of a moderate phenotype in the *HspB1*^-/-^ mouse was quite unexpected. Observation of *HspB1*^-/-^ mice over a period of 12 months did not reveal any signs of major morphological or physiological disturbance nor locomotion troubles or disease including neuropathy as predicted by Kim *et al*. [29]. Thus, Hsp27 on its own did not appear to be essential for development as also reported in mouse [30–32], fly [33], and zebrafish [34] but in contrast to a study by Eroglu *et al*. [35]. The moderate phenotype of the *HspB1*^-/-^ mouse indicates that the mutant has coped with the loss of Hsp27. This may be have been achieved through compensatory changes in the expression of cognate members of the sHsp family [2] sharing common functions with Hsp27 and transcriptionally regulated by HSF-1. Compensation which may be elicited by other small Hsps has probably occurred during early development and postnatal life. Consistently we previously reported changes in the status of sHsps in the muscles of the *HspB1*^-/-^ mouse [36, 37] that may support functional redundancy. However we report here specific characteristics of the *HspB1*^-/-^ mouse: a gender dimorphism with marked effects in males, an effect on body weight with no obvious changes in the growth rate, a lower plasma lipids profile, and an alteration of muscle ultrastructure. These gender differences agree with previous reports that the heat shock induction of *HspB1* is greater in males [38] and that Hsp27 regulates the activity [39] and expression [40] of the androgen receptor which plays a role in testosterone-related myogenesis [41].

Although Hsp27 is expressed at the highest levels of any tissues in muscle in basal condition [8], there was no specific apparent macroscopic or microscopic muscle phenotype associated with Hsp27 loss. There was no main impact of *HspB1* disruption neither on the relative weight of muscles nor on the size or types of muscle fibres. Similar observations were made in other vertebrate models, at the exception of the zebrafish for which Hsp27 was essential for optimal growth of craniofacial myofibres [34]. However, as we examined animals only postnatally we cannot exclude that the muscles of the mutant mouse could have developed slower than those of wild-type during foetal development. Interestingly, very specific differences between mutants and wild-type controls were detected at the protein level [37]. The electrophoretic profiles of m. *Soleus* proteins showed differences in myofibrillar proteins, especially the presence of a putative developmental MyHC isoform which remains to be confirmed. These differences may be explained by differences in the kinetics of myofibrillar protein expression between the *HspB1*^-/-^ mutant and the wild-type mouse during myogenesis. Thus they probably illustrate a slower development of mutant muscles as suggested above. Accordingly, the higher number of small fibres in the *HspB1*^-/-^ mouse may result from delayed muscle growth.

Most striking is the ultrastructure alteration of the skeletal muscles in the *HspB1*^-/-^ mouse. As a result of transgenic insertion to disrupt *HspB1* gene expression, introduction of *LacZ* gene via the targeting vector produces constitutive beta galactosidase expression. Albeit in the absence of substrate at the optimal pH *in situ*, this may be expected to compromise development or structure of tissues and organs. *LacZ* is broadly used as a reporter gene in transgenic studies with not any dedicated effect nor demonstrated cytotoxicity of beta galactosidase even after long term expression. Overexpression of this enzyme following injection or transgenic expression was demonstrated by immunocytochemistry in several studies while not any obvious change was detected in the heart and muscle cell ultrastructure *in vivo* [42–46]. However this does not disprove an effect of the *LacZ* reporter in the muscle ultrastructure in the HspB1 mouse but makes it very unlikely. We rather assume that change in muscle ultrastructure is a consequence of HspB1 invalidation. Indeed sHsps are important in maintaining cytoskeletal integrity [28, 47]. Especially during intense exercise and associated hyperthermia, high levels of sHsps are needed to prevent cellular damage in skeletal muscles [30]. Hsp27 interacts with key cytoskeletal protein elements and is associated with Z-disk maintenance [48]. This has been described at the level of microfilaments and intermediate filaments [49]. Under excise damage Hsp27 and αB-crystallin translocate from the intermyofibrillar space to the cytoskeletal/myofibrillar structures after a repeated bout of exercise [50]. They accumulate in myofibrillar structures, especially in the Z-line and intermediate filament structures, and in areas of disrupted sarcomeres. Cytoplasmic Hsp27 is thought to interact primarily with actin microfilaments [51], while cytoplasmic αB-crystallin appears most frequently to interact with intermediate filament proteins *e*.*g*. desmin [49]. Thus Hsp27 likely modulates stability, structure and dynamics of actin filaments [52]. In Zebrafish, localization of Hsp27 in resting length myofibrils correlates with regions adjacent to the Z-line. However in stretched myofibrils Hsp27 localization does not depend on desmin, α-actinin, myosin, or filamentous actin, but is connected with titin filaments [47]. A fine control of muscle architecture is of paramount importance during muscle development and myoblast differentiation, or when facing excessive damage. In *Xenopus* embryos lacking Hsp27 after morpholino injection, defects in myofibril architecture were observed in heart and skeletal muscle [53]. There were evidences of failure of myofibril assembly and sarcomere formation. Thus, the primary function of Hsp27 may be the regulation of myofibril structure. In our gene knock-out study, constitutive loss of Hsp27 also resulted in myofibril ultrastructural abnormalities in basal conditions as shown by disturbances in the cross-striation band pattern, deformation of myofibrillar structures, enlarged intermyofibrillar space, presence of some distorted mitochondria, and grouping of subsarcolemal mitochondria. Several hypotheses can be formulated to account for the muscle ultrastructural phenotype of the *HspB1* mutant mouse including misfolded proteins (loss of protective function through protein triage [54]), abnormal myofibrillogenesis and disorganization / loss of costameric proteins (e.g desmin) (loss of protective function from fragmentation) in the absence of Hsp27. Moreover, the alterations observed may be linked to altered maintenance of muscle homeostasis and activation of proteolysis through different mechanisms including proteolytic systems, apoptosis, proteasomal or autophagosomal protein degradation [55]. Interestingly, the specific phenotype of mutant muscle was associated with a lower abundance of desmin (a potential substrate of the calpain system and of caspase 3) and changes in the abundance of others proteins involved in muscle structure [36].

Since (i) under physiological conditions Hsp27 loss does not impair mouse development and physiology and (ii) the protein is stress-inducible, the phenotype of mouse is questioned under stress conditions. Indeed, the analysis of Hsp27 deficient muscle in stress conditions (exercise or hyperthermic challenge) will provide key elements on the function of Hsp27 and its interactors during pre- and post-natal development. This has been partly examined by Huang et al [56] who did not detect any change in the levels of several Hsps following hyperthermic challenge. However, as stated by these authors multiple sHsp targeted disruption might be helpful for further understanding of Hsps functional redundancy in mouse development.

## Conclusions

Overall, this study showed that mouse development and morphometric characteristics of their skeletal muscles were not impaired in the absence of the *HspB1* gene. These data suggest a functional redundancy between Hsps during development with subsequent apparently normal phenotype. However, the invalidation of the *HspB1* gene resulted in alteration of muscle ultrastructure. Therefore redundancy was not detected at ultrastructural levels suggesting a crucial role for Hsp27 in the regulation of protein make-up for muscle ultrastructure.

## Supporting Information

S1 Dataset Individual zootechnical and metabolic parameters of HspB1 mice and their control littermates. (XLS)
